# Mutualism and division of labour: a mutual expansion of concepts

**DOI:** 10.1098/rstb.2023.0266

**Published:** 2025-03-20

**Authors:** Jennifer H. Fewell, Judith L. Bronstein

**Affiliations:** ^1^ School of Life Sciences Arizona State University, Tempe, AZ 85287, USA; ^2^ Department of Ecology and Evolutionary Biology, University of Arizona, Tucson, AZ 85721, USA

**Keywords:** division of labour, mutualism, interspecific outsourcing, reciprocal outsourcing, cooperative sociality, levels of complexity

## Abstract

Division of labour within social groups and the interspecific relationships within mutualisms have traditionally been treated as separate research areas. In this opinion, we align terminologies and concepts between the two fields, by comparing within-group division of labour to the outsourcing of functions in mutualisms. Division of labour and interspecific outsourcing share fundamental similarities. Both are built from specialization of some individuals within the relationship on tasks or functions required for survival, growth and reproduction. Both also generate variable fitness outcomes. A key difference is that mutualisms generally generate direct fitness gain, while benefits from cooperative sociality often accrue from a mix of direct and indirect fitness. Additionally, the levels of physical and physiological specialization within many mutualisms expand far beyond the levels of differentiation seen in cooperative social groups, with the exception of reproductive division of labour. The consideration of between-species outsourcing in the context of division of labour allows expansion of our understanding of both fields and beyond, to consider general principles as drivers of division of labour, and role differences more broadly across levels of complexity.

This article is part of the theme issue ‘Division of labour as key driver of social evolution’.

## 1. Introduction

Snapping shrimp are famous for their ability to shoot a focused stream of water strong enough to stun or even kill an opponent. Some species of the genus *Synalphis* have evolved a specialist soldier caste that uses this weaponry to defend the snapping shrimp colony [1–3]. The presence of this specialist caste is an example of division of labour and is a factor in categorizing these species as eusocial [1,4]. However, the division of labour within this system is not limited to the shrimp colony alone. Colonies of eusocial *Synalphis* live within sea sponges*,* often specializing on one or a few sponge species as hosts [5]. In what has been categorized as a protection-based mutualism [6,7], the shrimp inhabitants defend the sponge against potential predators [7], while the sponge, in turn, provides shelter and protection for the shrimp [1,8]. The shrimp–sponge system thus involves a of division of labour both within and between species.

Moving from sea to land, wood-dwelling termites and the symbiotic protists living in their hindgut exemplify another system with close interdependence between taxa. Although termite social systems vary considerably [9,10], all are considered eusocial. Beyond their reproductive division of labour, task specialization within the colony is built primarily around defence [9,11]. Morphological soldier castes occur in multiple species [11], with soldier termites identified in samples as early as the Cretaceous [12].

All termites also rely on gut symbionts for cellulose digestion, while providing a protective environment for the microbes to survive. Termites can ingest but do not digest the cellulose-based products they consume; rather, the termite collects the resources, and their gut symbionts process them [13]. The symbiosis is mutualistic and obligate, with the phylogenies of wood-dwelling termites and their protists (superfamily Metamonada) showing close co-diversification [14]. The mutual outsourcing of protective and digestive functions between termite and gut symbiont is considered an important driver of social evolution in the Termatidae [13–17].

Each of the above examples illustrates a division of labour within a focal society that is also interwoven with a division of functions across species, a phenomenon we refer to as interspecific outsourcing. We argue that interspecific outsourcing can usefully be viewed as a division of labour in which different species specialize on different tasks. Just as workers within the termite and snapping shrimp colonies specialize on different roles, so do the individuals participating in the between-species relationship; social groups and species function together as a multilayered system of division of labour.

The two cases above differ in the fitness outcomes gained by outsourcing, just as dividing labour within a social group can generate differential fitness outcomes [18–21]. When the between-species relationship confers a mutual (reciprocal) fitness benefit we consider it a mutualism. In the shrimp–sponge outsourcing example, the shrimp colony gains clear benefit, while the fitness outcomes for the sponge are more context dependent, depending on predation levels [8]. Consequently, the shrimp–sponge association ranges from mutualism to commensalism. In contrast, the fitness gains in the obligate mutualism between the termites and protists are more certain.

Despite clear points of intersection between interspecific outsourcing and (intraspecific) division of labour, these phenomena have been addressed in two very different literatures. In this paper, we briefly review the concepts of division of labour and interspecific outsourcing from the perspectives of our different approaches—one of us as a social biologist interested in division of labour, and the other as an ecologist interested in the forms and functions of mutualistic relationships. We then present an argument of consilience, that certain types of mutualism are, effectively, interspecific divisions of labour. We argue that this perspective opens new questions and areas of inquiry at the intersection of these two well-studied phenomena, as well as at other levels of complexity such as multicellularity.

We explore division of labour and interspecific interactions involving outsourcing as extensions of the same concept: that specialization in an association—whether within or between species—can under limited circumstances lead to mutual fitness benefit. Within this framework, we examine why, when and how tasks are allocated to different species. We also consider how interspecific outsourcing can shape social organization within groups. Our ultimate goals in this essay are (i) to expand the understanding that within-species sociality is shaped by relationships that species have adaptively developed within the broader ecological communities in which they dwell and (ii) to consider the conditions under which interspecific relationships such as mutualism have shaped the ecology and evolution of division of labour within one or both species [22].

## 2. Division of labour as (intraspecific) cooperation

We define division of labour as occurring when different individuals within a group show non-random specialization on different tasks [23,24]. However, levels of specialization and their distribution within a group vary considerably across social contexts. It can be useful, therefore, to describe division of labour operationally as the degree to which different individuals in a group specialize on different tasks [24–26], with specialization defined as the degree to which an individual focuses on one task relative to other available tasks. Division of labour thus becomes a measurable group-level phenotype. As a phenotype, division of labour is not automatically adaptive, nor does it automatically confer a specific fitness benefit. It connects to cooperation when it is placed in the context of inclusive fitness outcomes [19–21,27–29]. Although these are generally positive in evolved cooperative systems, there are also contexts in which division of labour occurs emergently or spontaneously [30–32], or via manipulation or coercion [33,34] (and discussed below).

This being said, division of labour is intimately connected to cooperation, which we define as relationships within a social group in which individuals contribute to the group in ways that also bring some level of inclusive fitness to the performer(s) (building from [20,21]). Our definition has the implicit assumption of mutual benefit between cooperators but does not limit that benefit to direct fitness outcomes. This allows us to consider cooperation between species (mutualism) and cooperation within social groups, including those with reproductive division of labour. Cooperation occurs around context. It revolves around some specific set of needs where social performance can be beneficial. These generally fall into one or more of the following categories: defence, food collection and/or processing, offspring care and regulation of the environment (e.g. nest maintenance, thermoregulation and nest construction). Notably, these categories are also the bases of task specialization and division of labour.

Defining division of labour quantitatively produces a scale ranging from cases in which individuals show limited to no division of labour (they share in performance of all tasks), to those in which individuals fully specialize on one or a subset of tasks [25]. Thinking of division of labour in this way allows us to consider the broader range of contexts within which behavioural differences may play a role in shaping social organization. It also gives us a starting point to expand these comparisons beyond social groups or communities, to consider general principles for how differentiation in roles, whether social roles, ecological niches or cellular differentiation, may impact fitness across levels of organization [25,28,34–37].

If we consider the full range of social groups—both vertebrate and invertebrate—most division of labour is facultative and behavioural, including most of the non-reproductive division of labour seen within social insect colonies [26,38,39]. Division of labour includes instances of specialization at shorter time scales, such as when a foraging bee temporarily specializes on collecting pollen versus nectar [38,40], or when a cooperatively breeding pied babbler helper takes over feeding of a first brood, allowing parents to produce a second clutch [41]. More permanent relationships include the differentiated hunting roles seen in team-hunting species such as lions, bottle nose dolphins and other social predators, which are reinforced by experience and learning (e.g. [42,43]). Differences in behavioural roles, such as parental care or defence, can also be mediated by sex differences [44].

Division of labour within social groups also includes cases of morphological and associated physiological specialization that canalize an individual into a given role. It is important to emphasize, however, that this level of canalization may not be absolute and is generally limited to eusocial taxa. The most extreme example of morphological and specialization occurs with reproductive division of labour, in which workers have lost most of their ability to reproduce; indeed, reproductive division of labour is a defining characteristic of eusociality [45,46]. Morphological specialization outside of reproductive castes is often associated with group defence [47], snapping shrimp being one example, another being termite soldier castes that show head morphologies shaped specifically for defence [11,48].

There has been a tendency to focus discussion of division of labour around these more canalized roles, contributing to a communication barrier among researchers working on eusocial systems versus other cooperatively social species that has only recently begun to be bridged [19,49]. As examples, some workers of the leafcutter ant *Acromyrmex versicolor* show a propensity to specialize on removing trash [50]. Male helpers in campo flickers (*Colaptes campestris campestris*) disproportionately remove faecal sacs from the nest compared with females [51]. Traditionally, a social insect biologist would label these behaviours ‘tasks’ and might discuss them in the proximate context of individual specialization. Researchers focusing on cooperative breeding might discuss them as cooperative behaviours, with an emphasis on individual roles and fitness outcomes. Both are division of labour and cooperation.

As we discuss below, the issue of differences in language and perspective extends also to discussions about the relationship between interspecific outsourcing and social group division of labour, again highlighting the potential barriers that language can place on the exchange of ideas across fields. We suggest that reaching a level of consilience from within- to between-species associations could help bridge the gap, also for those of us studying social cooperation across social types and taxa.

## 3. Outsourcing, mutualism and division of labour

In parallel to the language of specialization and division of labour for within-species relationships, interspecific relationships have recently been discussed in the context of outsourcing [52–54]. In these relationships, individuals of a given species may outsource functions not to a certain set of individuals within their group but to a different species entirely. This often arises in cases where there are resources—critical goods or services—that organisms have difficulty acquiring on their own or within their conspecific group. Traits and behaviours then evolve that foster a long-term association with the resource-provider species. As with division of labour, the term ‘outsourcing’ identifies a relationship but does not in itself identify a specific fitness outcome for individuals of either species. When outsourcing is bidirectional and beneficial, it is considered a mutualism [22,55].

As for cooperative societies, species within mutualisms show varying levels of interdependency, from facultative to obligate [55]. Considering the many forms of interspecific outsourcing, three general classes of interspecific benefits have been recognized [55]. The first is acquisition of nutrients that would otherwise be inaccessible. Well-known cases include animals that gain critical carbohydrates by feeding on plants, including those that feed on floral nectar and on fruits, as well as plants in the family Fabaceae that procure fixed nitrogen from *Rhizobia*, soil bacteria that are housed in specialized root nodules. Without these critical resources, survival and reproduction are impossible or strongly curtailed. The relationship between termites and their gut symbionts also involves this category of interspecific benefit [13,14]. In an unusual case of nutrient outsourcing, the woolly bat, *Kerivoula hardwickii*, roosts in a pitcher plant, *Nepenthes hemsleyana*, whose hooded shape creates a space to rest cryptically. While roosting, the bats drop excreta into the funnel of the plant, providing a nitrogen-rich resource of predigested prey to the plant [52].

The second category of outsourced functions that produce benefits is protection from the biotic and/or abiotic environment. We began this paper with the shrimp–sponge association, in which sponges enlist shrimp as protectors; termite hosts similarly create a protective environment for the protists they house. Other well-explored interactions involve those in which plants and certain insects use ants to deter herbivores and predators, respectively [56,57]. Organisms that enlist protectors may either have no ability to defend themselves or have lost this ability.

The final benefit is transportation: organisms with limited ability to move enlist other species to transport them to suitable habitats. Examples range from anemones, which are moved among habitats by hermit crabs [58], to plants that use animals to move seeds to good spots to germinate. If seeds are not transported away from the parent plant, that plant is likely to experience extremely low reproductive success [59].

It is clear in these cases how and why the species that outsources a function will benefit. But why should organisms allow themselves to be used as a resource by another species? Unlike many of the cases discussed in the previous section, there is no indirect fitness benefit from doing so [60]. A very common answer is because the exploited species gains a critical resource in return, leading the interaction to be a mutualism. In mutualisms, outsourcing conveys reciprocal fitness benefits. Indeed, for this reason, mutualism is sometimes termed ‘reciprocal exploitation’.

Revisiting some of the examples above clarifies how reciprocal outsourcing works. A wide variety of insects, birds and mammals benefit from visiting flowers, as nectar provides carbohydrates and protein for fuelling critical life functions. Plants take advantage of these foragers’ need for food by outsourcing pollen transport to them; in their absence, pollen cannot move to conspecific flowers and reproduction will be strongly curtailed. Mutual advantage results from an exchange of nutrition for transportation outsourcing. Many plants gain protection from herbivores by enlisting ants as ‘biological warfare’ agents; the ants in turn profit because plants lure them with carbohydrate and protein-rich food (extrafloral nectar; [61–63]). In this case, the exchange involves protection and nutrition. Finally, leguminous plants outsource nitrogen acquisition to Rhizobium bacteria, and the bacteria in turn receive carbon in a form they are otherwise unable to obtain. Here again, mutual outsourcing functions for mutual benefit; it could equally well be termed reciprocal, interspecific division of labour.

Mutual outsourcing as division of labour (mutualism) almost always involves distantly related species, ones that occupy different trophic levels. This is likely because it usually arises in cases where there is no ability to obtain the critical resource from a conspecific or from a species with similar capacities [22]. For example, as only bacteria can synthesize certain amino acids, insects feeding on foods deficient in those amino acids must form persistent symbioses with them. On the other hand, some of the functions that are outsourced to mutualists can alternatively be obtained from the abiotic environment. For example, plants can be pollinated and have their seeds transported by the wind rather than by animals; aphids can be defended by chemicals rather than by ants.

Understanding the conditions under which interspecific outsourcing, whether reciprocal (mutualistic) or not, will arise and persist is a key question in evolutionary biology. There is extensive evidence in some cases for repeated evolutionary shifts between interspecific outsourcing and obtaining critical resources in other ways. In other cases, resources may be gained both by interspecific outsourcing and other means as well. For example, most ant-defended plants also have other means of defence, including spines, thorns or toxins [64]. When interspecific outsourcing will be part of a mixed resource-acquisition strategy and when it will be relied upon exclusively is an open question.

As illustrated by the examples above, mutualisms offer an expanded view of what division of labour/outsourcing can achieve beyond what we see in within-species cooperative groups. The morphological and physiological differences seen in mutualisms extend far beyond what is possible within a social group, with the exception perhaps of reproductive division of labour. They also offer an important contrast in how division of labour may evolve. Division of labour within intraspecific social groups generally does not begin de novo as a set of genetically based behavioural, physiological or morphological differences already in place. Although a division of labour can emerge via social interactions in the absence of selection [30–33], the resulting inclusive fitness benefits must be present as evolutionary drivers of increased task and morphological specialization [18,19,46]. In contrast, species within mutualisms bring to the relationship already evolved differences in form and function. These may be shaped further by the mutualism, but with few exceptions (e.g. [65]), they did not originate as its products.

Mutualisms and cooperative division of labour also differ in their selection drivers. The fitness benefit drivers of mutualism are most easily considered in terms of direct fitness outcomes (with acknowledgement that this may oversimplify the complex relationships when social groups are part of a mutualism, see below). In contrast, specialization within social groups can be driven by both direct and indirect fitness outcomes, with obligate division of labour and especially morphological specialization and loss of reproductive capacity, generally driven by indirect fitness outcomes [66–69].

## 4. Both division of labour and outsourcing vary in fitness outcomes

The issue of variation in levels of mutual benefit is a key theme in the studies of both mutualism [70] and cooperative sociality. Again, it is helpful in approaching this challenge to be clear in separating division of labour as trait from cooperation as relationship. In the ecological literature, language on outsourcing allows us to move from characterizations that conflate mechanism with fitness to ones that remain neutral about causation. We can then consider fitness impact via terms such as mutualism (mutually positive fitness benefits), versus parasitism (fitness benefit for one species at the expense of another), or commensalism (one species benefits at no measurable cost to the other)[71].

We can then consider the context of the relationship. Mutualisms are characterized as conferring mutual benefit in the different contexts of transportation, protection or nutrition. These overlap considerably with the contexts around which social cooperation evolves. Using the same logical principles for cooperative sociality helps us to recognize that the behaviours underlying cooperation are conceptually separate from their ultimate fitness consequences [19,23,24,29].

As in a mutualism, members of cooperative groups may vary in their individual fitness outcomes of cooperative division of labour while still gaining mutual (if not equal) benefit from the relationship [29,72]. However, specialization can also create significant cost disparities across tasks. In social groups, such cost disparities may lead to a system of task sharing in which individuals do not specialize on any given task [49]. As one potential example, in the purple crowned fairy wren (*Maurus coronatus*), social groups show limited to no division of labour; both subordinates and breeders share equally in tasks [73]. Similarly, analyses of meerkat social groups generally reveal little to no division of labour across the social group [74], although breeders can coerce helpers into performing specific tasks [75]. In sum, there are multiple paths between task organization and cooperation, from task sharing to specialization.

Interspecific outsourcing is framed specifically around specialization but also involves a range of fitness outcomes. When the benefit is mutually positive, it can be considered mutualism. However, the level of benefit varies with ecological context in almost every mutualism studied to date [76,77]. In the case of the snapping shrimp–sponge relationship, the benefit to the shrimp colony is clearly positive, while the impact on the sponge host varies depending on predation level [7]. In the presence of predaceous sea stars, *Synalphis* colonies can be beneficial to the sponge host; in the absence of similar predators, the presence of *Synalphis* is potentially neutral or even inhibitory to its growth [7,8].

The issue that task performance may bring differential fitness costs and benefits has been central to many multiple theoretical debates of within-species cooperation and cooperative sociality, particularly around direct fitness impacts (reviewed by [29]). Answers to the question of ‘why cooperate’ have placed various emphasis on mutual direct fitness benefit [21,78], direct benefit to the recipient (which becomes weak altruism [27]) and a focus on benefit to the social group while considering variation in fitness benefit to the actor [20,21].

Game theoretical models of cooperation, although they have produced important insights [79,80], do not completely resolve this issue [81]. This is in part because of the necessity to simplify parameters, often to dyadic relationships that have limited fit to the complexities of actual behaviour within social groups in nature [21]. Mutualisms often are based around relationship roles that are defined in function but have variable fitness relationships between the outsourcing species. This focus on relationships rather than individuals makes them perhaps an ideal comparison for game theoretical models of social cooperation.[82] Because kin selection is not an implicit component for interspecific relationships, these cases also may provide better parallels for non-kin cooperation, based on direct fitness outcomes.

## 5. When deception and coercion enter the relationship

As noted above, outsourcing a function to another species only becomes mutualistic if outsourcing has reciprocal benefit. Interspecific outsourcing may involve subterfuge rather than a mutually beneficial exchange [55,83,84]. In deceptive pollination systems, plants outsource pollen transfer to animals but offer no nectar in return: for example, they may resemble a nectar-rewarding species or a mate [84]. Other cases of unilateral interspecific outsourcing are exploitative in other ways. For instance, in nursing associations, one plant species outsources protection from the abiotic environment to a different plant species; however, that plant species can suffer from resource or light competition as a result [84]. Finally, interspecific outsourcing can be *commensal*; it can benefit the species that outsources a function but have no consequences, positive or negative, to its partner [85]. For example, vines exploit woody plant species to gain access to light environments, with minimal consequences for the host [85].

Sociality itself can create conditions that facilitate the exploitation of outsourcing, for example, via social parasitism [86]. An example of interspecific social parasitism is brood raiding (dulosis) by ants, in which raiding species invade nests and steal their brood to rear as workers in the parasitic nests [86–88]. Brood raiding occurs facultatively within some ant species when colonies infiltrate their neighbours for resources, including brood. Interspecific brood raiding ranges from similarly facultative cases to those species that rely completely on parasitized workers for all non-reproductive tasks; an extreme case of interspecific division of labour (Please note that we support arguments against using the historical colloquial language for interspecific brood raiding [89]). Ant nests also commonly include ‘guests’ or inquilines, individuals of other species, ranging from closely related ants to other arthropods, fungi and bacteria, that live in the nest without contributing to the colony but often also without clear negative impact [86,90].

Manipulation and coercion are recurring themes in the evolution of division of labour and in intraspecific sociality more generally [21,33,34,72,91,92]. Although they do not automatically confer differences in fitness [21,72], coercion into performing higher cost tasks can decrease fitness outcomes for the individual being coerced. In wasps, subdominant females that are pushed off the nest become foragers [93,94]. By being forced into this role, they lose the possibility of usurping the queen. Queens of the small carpenter bee, *Ceratina calcarata*, often under-provision one or more of her brood, constraining their development [95–97]. When they emerge as adults, these females are too small to compete as new queens. Instead of dispersing, they remain to help collect resources for the next generation of brood. Although this manipulation benefits the queen, it reduces the inclusive fitness benefit for the female helper [97]. These examples illustrate that, as with interspecific outsourcing, we need to identify the various fitness costs and benefits before categorizing them as mutualism, cooperation or potentially social parasitism.

## 6. Mutualism can direct social organization

Mutualism does not just offer an extension of our classification scheme for division of labour; it also expands our understanding of within-group social organization [22]. A central theme in the study of social behaviour is that within-group social interactions are shaped by ecological context. However, the question of how interspecific relationships such as mutualism serve as drivers of social organization is in the early stages of being answered. The relationships between the Termitidae and their gut symbionts, for example, are an ongoing focus of research, in part because termite sociality cannot be fully understood without consideration of this mutualism. However, our understanding of the complexity of these relationships is in a new period of acceleration [14]. A broader consideration of the reciprocal relationships between gut microbiomes and the social behaviour of their hosts is also rapidly expanding as a focus in social behavioral research [98–101].

The fungus-growing insects represent another context that illustrates how mutualism can drive within-group social organization. Mutualistic relationships in which insects cultivate fungus [102] are found across at least three prominent social insect groups, including the ambrosia beetles [103,104], the fungus-growing termites [13–15] and the leafcutter ants [105,106]. The mutualisms between social species in these taxa and their fungus are rich and evolutionarily long established [102].

The leafcutter ant’s fungus mutualism illustrates the intimacy of this connection. The relationship between leafcutter ants (*Atta, Trachymyrmex and Acromyrmex*; [105,106] and their fungi (*Leucoagaricus* spp.) is an obligate mutualism in which the fungus is nurtured by the ants, and the ants rely on the fungus as their primary (and for brood, virtually their only) food source. Ants and brood feed from nutrient-rich bundles of hyphae produced by the fungus and tended by the ants [107,108]. In producing the hyphae, the fungus takes a high-cellulose substrate (leaves) and converts it to a more accessible nutritional form for brood and ants [109].

For colonies of the desert leafcutter ant *A. versicolor*, a species found in the southern United States, assuring fungal survival and growth is a critical focus of task organization during colony initiation and early growth [110]. In this species, the risk of fungal loss is also the principle driver of primary polygyny, in which multiple unrelated ant queens form cooperative associations during colony founding that continue throughout the life of the colony [111,112]. Thus, a mutualism drives non-kin cooperation within this species.

The optimal conditions for fungus garden growth in leafcutters also create conditions beneficial to fungal pathogens [113,114]. Workers tend the fungus garden chemically, by applying metapleural gland excretions, and physically, by weeding infected areas and grooming out alien spores [113]. The fungus garden also uses a third layer of protection from pathogens via outsourcing to a bacterium (*Pseudonocardia*) that is maintained within the garden, and that confers resistance to a microbial parasite that infects both the ants and the fungus: a multilevel symbiosis [115,116]. In this case, the bacterium, fungus and ants are intertwined via their multiple mutualistic dyads. This example of ‘interactions between interactions’, also known as ‘multiple mutualism effects’ [117], illustrates how the relationships between social groups and mutualisms can be complex and multilayered.

The *Acromyrmex* leafcutters present an excellent example of how the mutualism between ants and fungus plays a major role in structuring division of labour within the colony. Fungal care is a significant part of the colony task repertoire, separate from foraging, nest maintenance and brood care [40,113]. The division of labour between within-nest fungal care and foraging is based in part on age-based polyethism, with older workers more likely to forage; it is also based on genetically based variation in task preferences among workers [40]. The division of foraging and fungal care into separate tasks likely helps limit contamination of the fungus garden by pathogens from outside of the nest.

In addition to being a focus of task specialization, the fungus also plays a central role in the communication network that regulates task organization, particularly in the context of foraging. Foraging leafcutter ants collect plant resources that are fed to the fungus. Foraging decisions are dependent on communication from the fungus. *Acromyrmex* foragers integrate the dietary experience of the fungus into the decisions they make when offered an array of suitable plant species to forage [118]. Foragers of the tropical genus *Atta* have a similar connection to their fungus. When given leaf matter treated to be unsuitable to the fungus (but undetectable to the foragers), they rapidly learn to avoid that plant species [119,120].

In other non-farming ant species, the selection of food items is primarily regulated around nutritional needs of the brood, which often require higher in protein–carbohydrate ratios than adults [121]. In *Acromyrmex*, however, foragers consistently select food items with low protein–carbohydrate ratios that most closely reflect fungus nutrient requirements [122,123]. The workers forage to feed the fungus. They do so even when brood levels and thus brood nutritional demands are increased [122]. For a leafcutter colony, the division of labour across tasks and the decisions made in performance of a task are so intertwined with their mutualism with fungi that understanding any aspect of colony function requires consideration of both the ants and fungus. A leafcutter colony is not merely an ‘ant’ colony; it is a mutualism.

## 7. Division of labour across levels of complexity: mutualisms to multicellularity

To keep within our scopes of expertise, we have limited our comparisons of division of labour to mutualism and social cooperation. However, this discussion could be expanded much more broadly to consider differentiation and division of labour across levels of complexity, from the role of outsourcing in the evolution of eukaryotes as mutualism to the evolution of multicellularity as division of labour.

**Figure 1.**
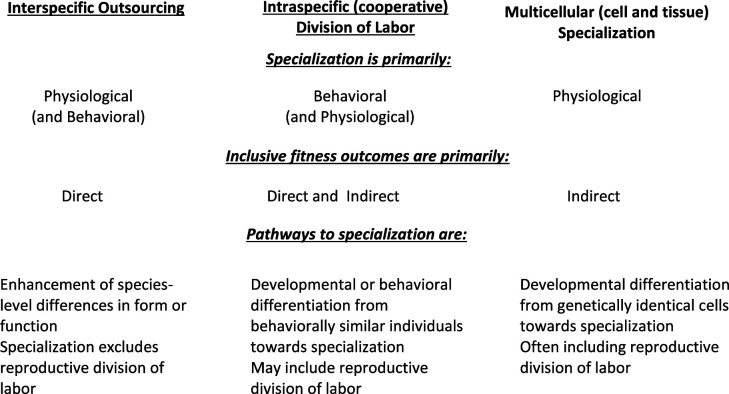
A simplified comparison of features for interspecific outsourcing in mutualisms, division of labour within cooperative social groups, and cell and tissue specialization within multicellular organisms. The comparison highlights major similarities and differences among the three levels of complexity, with acknowledgement that each level is more complex than outlined. The comparison illustrates the breadth of possibilities in types of specialized functions and their evolutionary drivers.

Multicellularity, cooperative sociality and mutualisms represent different levels of (or contexts for) complexity, but cooperation plays a central role in the evolution of all three. As for cooperative sociality and mutualisms, division of labour is a recurring theme in multicellularity, in that different individual subunits (cells, group members and species) take on different functions within the whole. The three vary, however, in the fitness drivers underlying the evolution of their cooperative relationships, and in the kinds of specialization seen within them (summarized in figure 1).

The evolutionary drivers for multicellularity, along with its associated cellular and physiological differentiation, have been fitted to both kin and multilevel selection models [68,69,124–126]; in both approaches, however, indirect fitness is the primary selection driver. In this way, they perhaps overlap most obviously with eusocial species. At the extreme, the obligate reproductive division of labour in highly eusocial taxa can be compared functionally (although not evolutionarily) to the somatic and gametic differentiation within multicellular organisms [37]. As with the evolution of eusociality, the evolutionary trajectory for division of labour in multicellular organisms begins with similarity in ‘group members’ and evolves to morphological/physiological differentiation as complexity increases.

That being said, the level of physiological differentiation within multicellular organisms expands far beyond that of any cooperative social system [92]. In this, they are matched by mutualisms. Indeed, mutualisms potentially surpass even tissue differentiation, because they involve different species bringing in diverse metabolisms, and often operating at different trophic levels. In contrast to multicellularity, however, direct fitness outcomes are the dominant selection driver for mutualism, although we note that symbiotic mutualisms and the evolution of eukaryotes would provide interesting points of discussion on this theme [124–126].

## 8. Conclusion

In this essay, we have highlighted potential points of consilience between division of labour within species and between-species relationships that involve outsourcing of critical life-history functions. We have expanded this discussion to consider how both division of labour and interspecific outsourcing vary in their impact on fitness, and how relationships incurring mutual benefit may be considered in the contexts of cooperation and mutualism. Hopefully, we have set the stage also for expanded discussion of how mutualism and cooperation intersect with other levels of complexity in the context of division of labour.

There are multiple points of consilience. The array of examples and studies on interspecific interactions—from commensalism to mutualism—can offer a rich perspective about the impact of mutual benefit outcomes on task performance across systems. Mutualisms expand the concept of what it means to divide labour within a cooperative system. The diverse kinds of outsourcing across species and trophic levels in mutualisms serve as exemplars of how far division of labour can go. As such, they have the potential to expand our understanding of division of labour beyond the scales seen and understood for within-species cooperative systems.

Extreme morphological specialization occurs rarely within social systems, and when present it is generally associated with indirect fitness benefit. Mutualisms are thus free of entanglements regarding direct versus indirect fitness, a long-standing issue in discussions of cooperative sociality. This makes them potentially useful comparisons for non-kin-based social cooperation and associated game theoretical models. In turn, the rich discussions of how division of labour and social cooperation intertwine within social groups offer new ways to consider mutualisms as both ecological and social relationships. The deep dives in social biology on how cooperation links to fitness, both theoretical and empirical, serve as points of consideration for mutualisms in the ecological realm. How does our understanding of the complexity of cooperation and fitness within social groups map onto the complex relationships found within mutualisms? What can the exploration of each realm teach us about the other? With these questions, we hope the discussion will continue.

## Data Availability

This article has no additional data.
